# Antibiotic skin testing accompanied by provocative challenges in children is a useful clinical tool

**DOI:** 10.1186/1710-1492-9-22

**Published:** 2013-06-14

**Authors:** Fotini D Kavadas, Anna Kasprzak, Adelle R Atkinson

**Affiliations:** 1 Section of Clinical Immunology and Allergy, Department of Pediatrics, Alberta Children’s Hospital and University of Calgary, 2888 Shaganappi Trail NW, Calgary, Alberta T3B 6A8, Canada; 2 Division of Clinical Immunology and Allergy, Department of Pediatrics, The Hospital for Sick Children, and University of Toronto, 555 University Ave. Rm7279 Elm/Burton wing, Toronto, Ontario M5G1X8, Canada

**Keywords:** Antibiotic allergy, Drug testing, Skin testing, Provocative challenge

## Abstract

**Background:**

Diagnostic testing to antibiotics other than to penicillin has not been widely available, making the diagnosis of antibiotic allergy difficult and often erroneous. There is often reluctance in performing challenges to antibiotics when standardized testing is lacking. However, while the immunogenic determinants are not known for most antibiotics, a skin reaction at a non-irritating concentration (NIC) may mean that antibodies to the native form are present in the circulation. While the NIC’s for many non penicillin antibiotics have been determined in adults, the use of these concentrations for skin testing pediatric subjects prior to provocative challenge has not been done. Our objective was to determine if we could successfully uncover the true nature of antibiotic allergy in children using these concentrations for testing.

**Methods:**

Children were included between 2003–2009 upon being referred to the Drug and Adverse Reaction/Toxicology (DART) clinic of the Hospital for Sick Children in Toronto, Ontario Canada. The referral needed to demonstrate that clinical care was being compromised by the limitation in antibiotic options or there was a significant medical condition for which the label of antibiotic allergy may prove detrimental. Patients were not seen if there was a suggestion of serum like sickness, Stevens Johnson Syndrome or Toxic Epidermal Necrolysis. Patients were excluded from testing if there was objective evidence of anaphylaxis. All other patients were consented to receive testing and/or challenges. A retrospective chart review was then performed of the results.

**Results:**

We were able to exclude an antibiotic allergy in the majority of our patients who had a negative intradermal test result and were then challenged (>90%). Only one patient was challenged with a positive intradermal test to Cotrimoxazole because of a questionable history and this patient failed the provocative challenge. While we did not challenge more patients with positive testing, we did note that 10/11 (91%) patients with positive intradermal testing had some aspect of a Type 1 reaction in their history.

**Conclusions:**

Through testing with NIC’s of various antibiotics in children and providing provocative challenges based on negative skin testing results, we were able to advance the medical care of the majority of our patients by increasing their antibiotic options in order to successfully treat future infections. While challenging patients with positive testing was not deemed ethically appropriate at this stage of our study, it would be a useful future step to reaching statistical validity of testing to these antibiotics.

## Background

The label of drug allergy is a commonly encountered problem in medical practice. As documented with penicillin allergy, the accuracy of such labels is often poor and the vast majority is actually not allergic to the antibiotic in question [1-3]. If the individual is most often well and the sensitivity profile of the particular microorganism is expansive, these drug allergy labels may not prove detrimental to successfully treating an infection. Elucidating the precise nature of the drug allergy label becomes more critical in the case of a complex medical condition predisposing to recurring infections, multiple drug allergy or when targeting an organism with a very narrow sensitivity profile. Certainly in an era where multiple drug resistant strains of bacteria (so called ‘superbugs’) have caused outbreaks of deleterious infection, a label of just one antibiotic allergy may become relevant even for otherwise healthy individuals.

Unfortunately, standardized drug allergy testing is only available commercially for penicillin because the clinically relevant metabolic byproducts are known. Even in this case, it is difficult to obtain a mixture of minor determinants which can cause a substantial proportion of reactions. Not knowing the metabolic determinants that are clinically relevant for other antibiotics has been a limiting factor in the production of testing materials. Furthermore, the concentration of antibiotic that is appropriate for triggering an IgE mediated reaction and not an irritative reaction during skin testing has been a subject of debate and one that has limited the scope of antibiotic allergy testing. However, Empedrad et al., through sequentially testing various concentrations of certain non penicillin antibiotics in adults, were able to determine many non-irritating concentrations (NIC’s) [4]. Ultimately, however, drug provocation is the gold standard in definitively excluding an IgE mediated reaction to the drug [5]. The reassurance of negative skin testing prior to a provocation can be profound both for patients and for health practitioners who must justify readministration of these medications [1,3,5]. Since these particular NIC’s used in adults (Table 1) had never been trialed in children prior to a provocative challenge, we hypothesized that the NICs would likely be similar in the younger population. In this scenario, we believed it would be a useful tool to help decide which patients would be suitable to proceed to a drug challenge. We believed that the impact in a tertiary or quaternary care centre in terms of expanding treatment options for complex patients had the potential of being significant and worth the risk of a reaction during a challenge if patients were chosen carefully.

**Table 1:** Antibiotics included in our testing panel

**Antibiotics in our testing panel**	**Skin prick testing conc. (mg/ml)**	**Intradermal testing conc. (mg/ml)**	**Number of patients**	**Percent of total**
Cotrimoxazole	80	0.8	12	20.3%
Ceftriaxone	100	10	8	13.6%
Cefuroxime	100	10	7	11.9%
Clindamycin	150	15	7	11.9%
Cefazolin	330	33	6	10.2%
Ceftazidime	100	10	5	8.5%
Azithromycin	100	0.01	5	8.5%
Erythromycin	50	0.05	3	5.1%
Aminoglycoside	40	4	2	3.4%
Vancomycin	50	0.005	2	3.4%
Levofloxacin	25	0.025	2	3.4%

## Methods

Our study was approved through the Research and Ethics Board of the Hospital for Sick Children. Visits to our Drug Adverse Reaction and Toxicology (DART) clinic were screened between 2004–2009 for a history of possible allergy to various antibiotics. Since this was only meant as an introductory study, we accepted children up to 18 years of age into our clinic if the referral demonstrated that the patient had a complex medical condition predisposing to multiple infections, if medical treatment was significantly compromised by the limitation in antibiotic options or if there was limited organism sensitivity. Table 2 outlines the range of disorders found in our cohort. We believed that the clinical impact during this introductory phase of our study was highest for these patients. Healthy children not experiencing frequent infections were typically not even brought to the clinic even if referred. Also, if the referral was even remotely suggestive of serum like sickness, Stevens Johnsons Syndrome or Toxic Epidermal Necrolysis, the patient was not brought to clinic.

**Table 2:** Medical conditions of patients included for testing

Limited treatment options/Reaction to multiple antibiotics	12 (28.6%)
Chronic Respiratory Disease	9 (21.4%)
	Cystic fibrosis: 7
	Primary ciliary dyskinesia: 1
	Asthma: 1
Asplenic	6 (14.3%)
	Sickle Cell: 1
	Hereditary Spherocytosis: 1
End Stage Renal Disease	6 (14.3%)
Muskoloskeletal Infection/Foreign Body	5 (11.9%)
Severe ureteric reflux	2 (4.8%)
Spina Bifida	1 (2.3%)
Malignancy	1 (2.3%)

During the initial visit, each patient and their family were interviewed with a standard questionnaire that was meant to extract the type of antibiotic that caused the reaction and the indication for its administration. A detailed history around the reaction to the medication was elicited and past medical and pharmacy records were reviewed in detail to minimize recall bias. We also strived to appreciate how challenging it subsequently became to treat that particular infection or future infections. For example, if a cystic fibrosis patient became labeled with a Ceftazidime allergy, there would be a limitation on treating Pseudomonas infections which cause significant morbidity and mortality in this population. Patients were excluded from proceeding with testing if there was a very clear history of anaphylaxis as documented by patient history but especially corroborated by medical records. The selected patients were skin tested with NICs published for adults by Empedrad et al. and outlined in Table 1[4]. Histamine was the positive control for skin prick testing and 0.9% saline was the negative control for both skin prick and intradermal testing. A provocative challenge with the antibiotic was offered in either the oral or intravenous form if intradermal testing was negative or if there was sufficient doubt about a positive result. Patients were in general not challenged if they had a positive intradermal test during this phase of our study. Due to the young age of some of our patients who would have a limited ability to articulate the onset of severe adverse symptoms, our ethics board believed that the level of risk was too high to administer medications in this setting.

## Results

**Figure 1:**
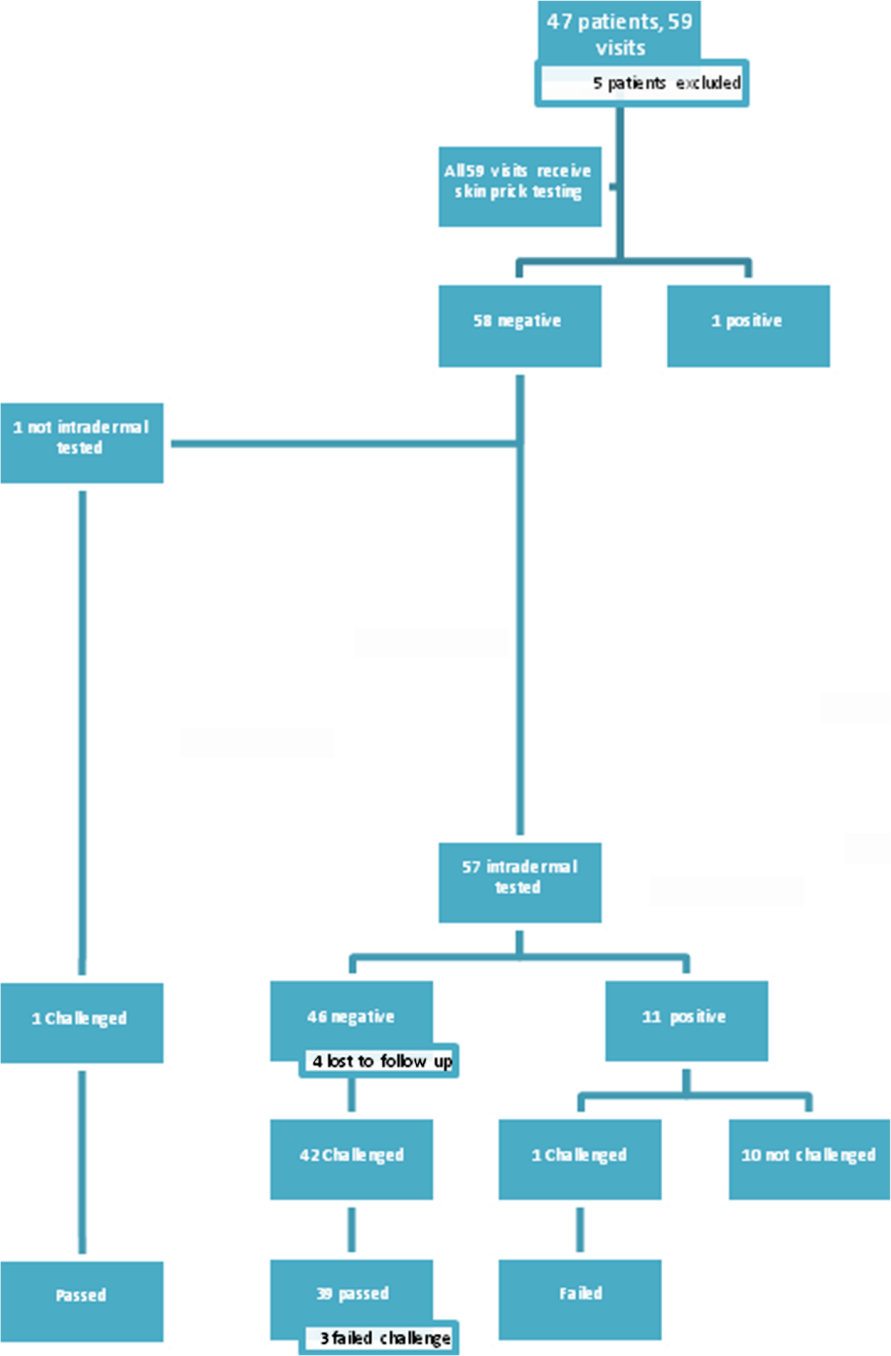
Summary of results of patient testing and challenges.

We examined a total of 47 patients in our clinic and 42 were included in the study. The five that were excluded had a history very convincing for true anaphylaxis and had alternatives for treatment. The medications they reacted to included Ceftriaxone (3 patients) and Cefuroxime (2 patients). Some patients were seen for testing to more than one antibiotic making the total number of visits 59. For a summary of the patients’ medical conditions and results, please refer to Table 2 and Figure 1.

At all 59 visits skin prick testing was performed. Only one patient had positive skin prick testing. This patient had a history of possible anaphylaxis with Ceftriaxone administration and was therefore not tested via the intradermal route. On 57/59 (96.6%) visits, intradermal testing was performed. One other patient did not have intradermal testing because behaviour did not permit this to be done safely.

46/57 visits (80.7%) tested with the intradermal method had negative testing while 11 were positive (19.3%). The majority of the latter, 10/11 (91%), had a history consistent with an immediate IgE mediated reaction either to the antibiotic in question or to an antibiotic in the same class. The majority of the positive tests were accounted for by a history of sensitivity to Cotrimoxazole, 6/11 (54.5%). One patient had sensitivity to Erythromycin, 1 to Vancomycin, 1 to Ceftriaxone and 2 to Azithromycin.

42 of the 46 patients (91.3%) with negative intradermal testing went on to receive an oral challenge with the antibiotic that was tested. Attempts were made to challenge the other 4 patients with negative testing but they did not show to their challenge appointments and were lost to follow up. One patient with positive intradermal testing to Cotrimoxazole was challenged due to an unusual history of only hypotension with the medication. It was thought that perhaps sepsis may have been the cause of his hypotension with the initial administration. Another challenge was done with parental consent without intradermal testing because of aggressive patient behaviour.

Of the 44 total challenges performed, 40 were successful (90.9%) after a one hour observation period and did not have a delayed reaction after being discharged. 39/42 (92.9%) of patients with negative intradermal skin testing tolerated the medication.

The 4 failed challenges including the patient with positive intradermal testing to Cotrimoxazole. The other 3 patients failed despite a negative intradermal test. However, in their referral histories, one had hives with Tobramycin, and another had vomiting and pharyngeal pruritus with Ceftriaxone. The third had a maculopapular rash in the referral history but had urticaria 4 days after our challenge to Cefuroxime.

23 patients experienced hives without anaphylaxis in the original history. All had negative skin prick testing. However, 18/23 (78.3%) had negative intradermal testing and 5 patients (21.7%) tested positive. 17 of the 18 patients with negative intradermal testing were challenged. One patient did not come to the challenge appointment. 16/17 (94.1%) of those with negative skin testing but urticaria in the history passed the challenge.

Alternatively, the majority of patients with a history of possible anaphylaxis tested positive. 4/6 (66.7%) had positive skin testing and 1 with negative testing failed a provocative challenge. The other patient with negative testing to Ceftriaxone did not appear for the provocative challenge.

On 15 visits, we tested to an alternative antibiotic other than in the referral history in order to give the patient a treatment option. 9 of these patients were tested to an antibiotic in a different class and tolerated the challenge. 6 patients were tested with an antibiotic in the same class and 2 of these patients had positive intradermal testing (1 cephalosporin and 1 macrolide). The remaining 4/6 patients tolerated the challenge to an antibiotic in the same class.

## Discussion

NIC’s for testing to a limited list of antibiotics have been validated in adults [4]. We believed that testing with these concentrations could be a useful decision making tool in performing provocative challenges that would definitively characterize antibiotic allergies in our pediatric patients. While standardized testing to a wide range of antibiotics is lacking, elucidating antibiotic allergies in the pediatric population can have a major clinical impact as this population tends to experience a high burden of infectious diseases while their immune systems mature. The vast majority (10/11, 90.9%) of our patients who had positive testing also had a history of an immediate IgE mediated reaction such as urticaria. Over 90% of those with negative skin testing were able to tolerate a provocative challenge to the medication. Since we used the intact antibiotic for testing rather than metabolites, our promising findings suggest that there may in fact be IgE antibodies to the native drug itself rather than only to immunogenic determinants or unique haptens formed after metabolism as previously thought [1,4]. We were able to improve the care of patients with chronic medical conditions by uncovering falsely diagnosed allergy to antibiotics. In the case of cystic fibrosis patients, having an expanded repertoire of antibiotics to treat aggressive organisms such as Pseudomonas could prove life altering. Treating febrile neutropenia with the least toxic regimen of antibiotics may also make a significant difference to clinical outcome in a cancer patient. Furthermore, we have demonstrated that using only a history of minor acute Type 1 mediated symptoms may not be adequate to diagnose antibiotic allergy. In penicillin testing, the history has been found to be poorly predictive of subsequent skin test results [3,6-8]. Symptoms such as urticaria may be triggered by other causes such as the underlying infection itself [9]. In our study, most patients with urticaria alone who did not have associated anaphylactic symptoms had negative testing and passed the provocative challenge.

A significant limitation of our study is that we did not challenge patients with urticaria in the history who had positive skin testing. This would certainly have given greater impact to the meaning of a positive test and would have allowed us to be even more confident that a positive result is not merely an irritative effect in a child. While we are at ease in our clinic with the caution and technique we use when challenging patients that have a higher risk of reacting, we could not obtain ethics approval for testing all children with positive testing. Now that our outcomes of challenges with negative testing are favourable in the sense that we did not substantially under identify the number of truly allergic children, we hope to obtain permission to challenge all patients, regardless of test result in the future. We also recognize that the numbers of children tested to each antibiotic were relatively low and we hope to significantly increase these numbers going forward. Furthermore, we have assumed that the NIC’s shown in adults are transferable to children. Verifying this further would require intradermal testing children to multiple concentrations and performing the same on controls without a history of drug allergy. Convincing parents to consent to intradermal testing multiple times when they may already be struggling with a chronically ill and needle phobic child would be a difficult task and one that our ethics board was not open to entertaining.

## Conclusions

Skin testing to multiple different antibiotics in their native form in children can potentially be a reliable, objective guide to successful challenges. In a tertiary or quaternary care centre, an allergy service that is willing to take on the risk involved in challenging patients with a wide array of antibiotics that may have caused a reaction in the past can make a significant contribution to the care of complex patients.

## Abbreviations

NIC: Non irritating concentration; DART: Dart and Adverse Reaction and Toxicology Clinic.

## Competing interests

The authors declare that they have no competing interest.

## Authors’ contributions

FK contributed to the assessment of patients and the collection, interpretation and presentation of data and all stages of drafting of this manuscript. AK contributed to monitoring patients during testing and challenges, collecting patient data and following up their clinical status. AA conceived of the study, contributed to study design, supervised assessment of patients and revised this manuscript. All authors read and approved the final manuscript.

## References

[B1] Gruchalla RS Drug allergy J Allergy Clin Immunol 2003 111 S548 S559 10.1067/mai.2003.93 12592301

[B2] Gruchalla RS Pirmohamed M Clinical practice: antibiotic allergy N Engl J Med 2006 354 601 609 10.1056/NEJMcp043986 16467547

[B3] Romano A Demoly P Recent advances in the diagnosis of drug allergy Curr Opin Allergy Clin Immunol 2007 7 299 303 17620820 10.1097/ACI.0b013e328216f4d4

[B4] Empredrad R Darter AL Earl HS Gruchalla RS Nonirritating intradermal skin test concentrations for commonly prescribed antibiotics J Allergy Clin Immunol 2003 112 629 630 10.1016/S0091-6749(03)01783-4 13679828

[B5] Aberer W Bircher A Romano A Blanca M Campi P Fernandez J Drug provocation testing in the diagnosis of drug hypersensitivity reactions: general considerations Allergy 2003 58 854 863 10.1034/j.1398-9995.2003.00279.x 12911412

[B6] Wong BB Keith PK Waserman S Clinical history as a predictor of penicillin skin test outcome Ann Allergy Asthma Immunol 2006 97 169 174 10.1016/S1081-1206(10)60008-7 16937746

[B7] Jost BC Wedner HJ Bloomberg GR Elective penicillin skin testing in a pediatric outpatient setting Ann Allergy Asthma Immunol 2006 97 807 812 10.1016/S1081-1206(10)60973-8 17201241

[B8] Torres MJ Blanca M Fernandez J Romano A Weck A Aberer W Diagnosis of immediate allergic reactions to beta-lactam antibiotics Allergy 2003 58 961 972 10.1034/j.1398-9995.2003.00280.x 14510712

[B9] Thong BYH Blanca M Risk factors and diagnostic tests in drug allergy Curr Opin Allergy Clin Immunol 2007 7 297 298 10.1097/ACI.0b013e32825fea26

